# The Candida glabrata Parent Strain Trap: How Phenotypic Diversity Affects Metabolic Fitness and Host Interactions

**DOI:** 10.1128/spectrum.03724-22

**Published:** 2023-01-12

**Authors:** Jane Usher, Gabriela F. Ribeiro, Delma S. Childers

**Affiliations:** a Medical Research Council Centre for Medical Mycology, University of Exeter, Exeter, United Kingdom; b University of Aberdeen, Institute of Medical Sciences, Aberdeen Fungal Group, Aberdeen, United Kingdom; University of Iowa Hospitals and Clinics

**Keywords:** *Candida glabrata*, phenotype, strains, metabolism, cell wall, host interactions, virulence

## Abstract

Reference strains improve reproducibility by standardizing observations and methodology, which has ultimately led to important insights into fungal pathogenesis. However, recent investigations have highlighted significant genotypic and phenotypic heterogeneity across isolates that influence genetic circuitry and virulence within a species. Candida glabrata is the second leading cause of candidiasis, a life-threatening infection, and undergoes extensive karyotype and phenotypic changes in response to stress. Much of the work conducted on this pathogen has focused on two sequenced strains, CBS138 (ATCC 2001) and BG2. Few studies have compared these strains in detail, but key differences include mating type and altered patterns of expression of EPA adhesins. In fact, most C. glabrata isolates and BG2 are *MATa*, while CBS138 is *MATα*. However, it is not known if other phenotypic differences between these strains play a role in our understanding of C. glabrata pathogenesis. Thus, we set out to characterize metabolic, cell wall, and host-interaction attributes for CBS138 and BG2. We found that BG2 utilized a broader range of nitrogen sources and had reduced cell wall size and carbohydrate exposure than CBS138, which we hypothesized results in differences in innate immune interactions and virulence. We observed that, although both strains were phagocytosed to a similar extent, BG2 replicated to higher numbers in macrophages and was more virulent during Galleria mellonella infection than CBS138 in a dose-dependent manner. Interestingly, deletion of *SNF3*, a major nutrient sensor, did not affect virulence in G. mellonella for BG2, but significantly enhanced larval killing in the CBS138 background compared to the parent strain. Understanding these fundamental differences in metabolism and host interactions will allow more robust conclusions to be drawn in future studies of C. glabrata pathogenesis.

**IMPORTANCE** Reference strains provide essential insights into the mechanisms underlying virulence in fungal pathogens. However, recent studies in Candida albicans and other species have revealed significant genotypic and phenotypic diversity within clinical isolates that are challenging paradigms regarding key virulence factors and their regulation. Candida glabrata is the second leading cause of candidiasis, and many studies use BG2 or CBS138 for their investigations. Therefore, we aimed to characterize important virulence-related phenotypes for both strains that might alter conclusions about C. glabrata pathogenesis. Our study provides context for metabolic and cell wall changes and how these may influence host interaction phenotypes. Understanding these differences is necessary to support robust conclusions about how virulence factors may function in these and other very different strain backgrounds.

## INTRODUCTION

Historically, reference and type strains have provided important mechanistic insights into host-pathogen interactions. For example, studies using Candida albicans SC5314 led to major breakthroughs in understanding biofilm formation, virulence factor expression, and the role of the fungal toxin candidalysin in epithelial cell interactions (1–5). However, early studies hinted that significant genotypic-phenotypic variation existed in C. albicans clinical isolates, leading to a broad range of virulence outcomes in murine systemic infection models (6). More recent work interrogated multiple C. albicans clinical isolates and discovered genetic circuit diversification in the key transcriptional pathways regulating biofilm formation, demonstrating that specific transcriptional control of biofilm formation was largely strain dependent (7). Intraspecies diversity has also been observed in C. albicans antifungal tolerance to fluconazole (8). Therefore, while model strains have served a vital role in understanding mechanisms driving pathogenesis, long-term *in vitro* reliance on these systems has limited our understanding of intraspecies diversity and the role this plays in microbial evolution and pathogenesis.

C. albicans is an important human fungal pathogen and the leading cause of candidiasis worldwide. However, the prevalence of infections caused by non-*albicans Candida* species has been increasing over the last number of decades, and some species, such as Candida glabrata and Candida auris, are associated with high levels of antifungal and multidrug resistance.

C. glabrata is the second leading cause of candidiasis in North America and Europe and can rapidly develop resistance to azole and echinocandin drugs. It is responsible for up to 40% of *Candida* species bloodstream infections (9–12), with increasing mortality rates and an increased resistance to antifungal therapies, specifically azoles, mostly due to its plastic genome (13–15). Unlike many *Candida* species, bloodstream isolates of C. glabrata from the same patient can have different karyotypes (14–16). For example, extra chromosomes carrying large (120 to 420 bp) segmental duplications were found in clinical isolates of C. glabrata (15, 16). These supernumerary chromosomes contain centromeric regions and have acquired telomeric regions but exhibit reduced genetic stability. Such a mechanism of gene dosage instability is a potentially effective strategy to overcome environmental stresses by altering phenotypic responses (14, 17–19). Thus, genotype plasticity may facilitate rapid pathogen adaptation and pathogenicity by producing transient variants with altered abilities to evade and survive the challenges presented in different host niches (20).

C. glabrata strains CBS138 (ATCC 2001) and BG2 are often used for *in vitro* studies. BG2 is a derivative strain from the parental strain B, which was a fluconazole-resistant vaginitis clinical isolate (21). Studies with BG2 have been instrumental in identifying novel adhesins (22, 23) and other virulence factors, such as secreted aspartyl proteases (24–27), involved in C. glabrata virulence. CBS138, on the other hand, was isolated from human feces and was the first C. glabrata strain to have its genome fully sequenced (28, 29). A deletion collection spanning approximately 12% of the C. glabrata genome is available in the CBS138 background and has been used to identify genes involved in antifungal tolerance and macrophage survival (30, 31). Although BG2 and CBS138 dominate C. glabrata research, their comparative genotypic and phenotypic diversity and subsequent roles in pathogenesis remain poorly understood.

In the manuscript, we set out to characterize the phenotypic differences between these two commonly used C. glabrata isolates and establish how their phenotypic diversity influences our understanding of C. glabrata pathogenesis. We performed Omnilog metabolic profiling, which identified important differences in carbon and nitrogen source utilization between these two strains, particularly for methionine and tryptophan. In addition, we investigated the structure and carbohydrate exposure of the cell wall, established how each strain survives macrophage challenge, and determined their capacity to cause disease in a Galleria mellonella larval infection model. Collectively, these data provide essential phenotypic insights into improve our understanding of the diversity driving C. glabrata pathogenesis in important reference strains.

## RESULTS

### BG2 and CBS138 have distinct metabolic profiles.

**FIG 1:**
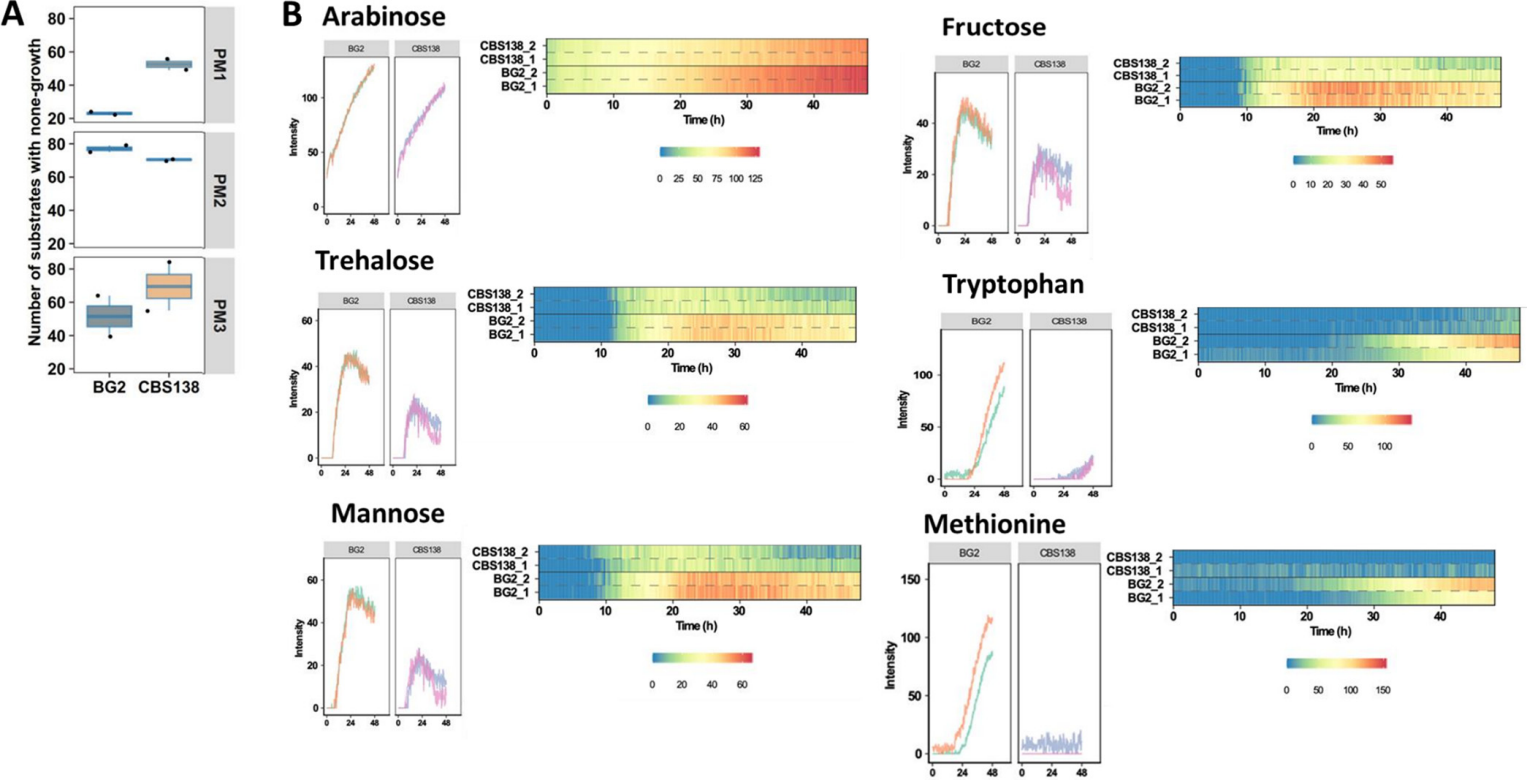
BG2 and CBS138 have distinct metabolic profiles for carbon and nitrogen utilization. (A) General overview of metabolism differences between BG2 and CBS138 as measured on the Omnilog system. Plates PM1 and PM2 are individual carbon sources, and PM3 has individual nitrogen sources. A full detailed list of the different carbon and nitrogen sources is shown in Fig. S1 and S2 in the supplemental material. (B) Representative images of some of the key carbon and nitrogen sources with different utilization profiles between BG2 and CBS138. For arabinose, a very short lag time and high growth rate are observed, more so in BG2 than CBS138. In trehalose, there is an observed long lag time with a high growth rate that quickly reaches stationary phase in BG2. With mannose, there is a medium lag time and growth rate, with BG2 quickly reaching stationary phase, which was also observed for growth in fructose. For growth in tryptophan, a medium lag time followed by a high growth rate was observed for BG2, with neither strain reaching stationary phase in the 48-h run. In methionine, a clearly different lag time and growth rate are observed for both strains, with BG2 not showing any significant growth until after 20 h.

Metabolism underpins yeast physiology, stress adaptation, and the capacity to cause disease. To detect differences in the metabolism of BG2 and CBS138, we performed metabolic profiling using the Omnilog system, which measures growth phenotypes in the presence of different metabolites (32). We examined cell growth on a series of carbon and nitrogen sources (Fig. 1; see Fig. S1 and S2 in the supplemental material). Our results indicate significantly different growth phenotypes between BG2 and CBS138 when growth was limited to specific carbon or nitrogen sources. We observed that BG2 was more efficient at metabolizing different carbon sources when in a glucose-limited environment (Fig. 1A and B). This difference included fermentable carbon sources, with significant differences in arabinose consumption where BG2 had a short lag time and a high growth rate compared to CBS138 (Fig. 1B). In trehalose, a sugar composed of two glucose molecules, BG2 had a long lag time with a high growth rate that quickly reached stationary phase. Further, during mannose and fructose utilization, the two sugars most preferred by yeast after glucose (33), BG2 had a medium lag time and growth rate that quickly reached stationary phase (Fig. 1B). The ability to utilize carbon sources apart from glucose is a trait necessary for many pathogens when faced with changes in environment such as in blood versus mucosal membranes.

### BG2 has a growth advantage on tryptophan and methionine.

**FIG 2:**
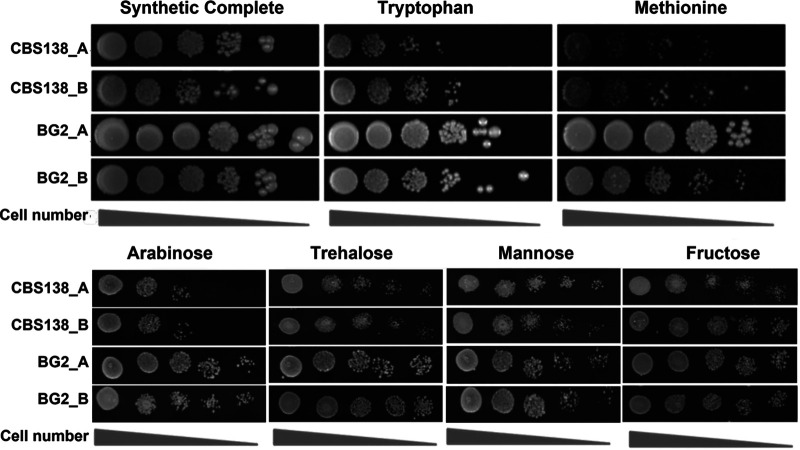
Spot dilution assays confirm differences in carbon and nitrogen source utilization between BG2 and CBS138. Serial dilutions of cells starting from 10^5^ cells/mL were spotted onto the indicated plates and incubated at 37°C for 24 h.

We noted that when screened on different nitrogen sources, a different phenotype was observed for BG2 when grown on tryptophan and methionine compared to that of CBS138 (Fig. 1). On tryptophan, BG2 had a medium lag time followed by a high growth rate; however, neither strain reached stationary phase over the course of this experiment (48 h). When tested on solid media, we observed that CBS138 had a growth defect in the presence of tryptophan compared to BG2 after 24 h at 37°C (Fig. 2).

Under growth in the presence of methionine, a different lag time for both strains was observed, with no significant CBS138 growth during the 48-h period, whereas BG2 did not show any significant growth until after 20 h (Fig. 1). Similarly, when screened on solid media, we observed a strong attenuation in growth of CBS138 on methionine media compared to BG2 (Fig. 2).

### Cell wall carbohydrates differ in structural complexity and abundance between strains.

**FIG 3:**
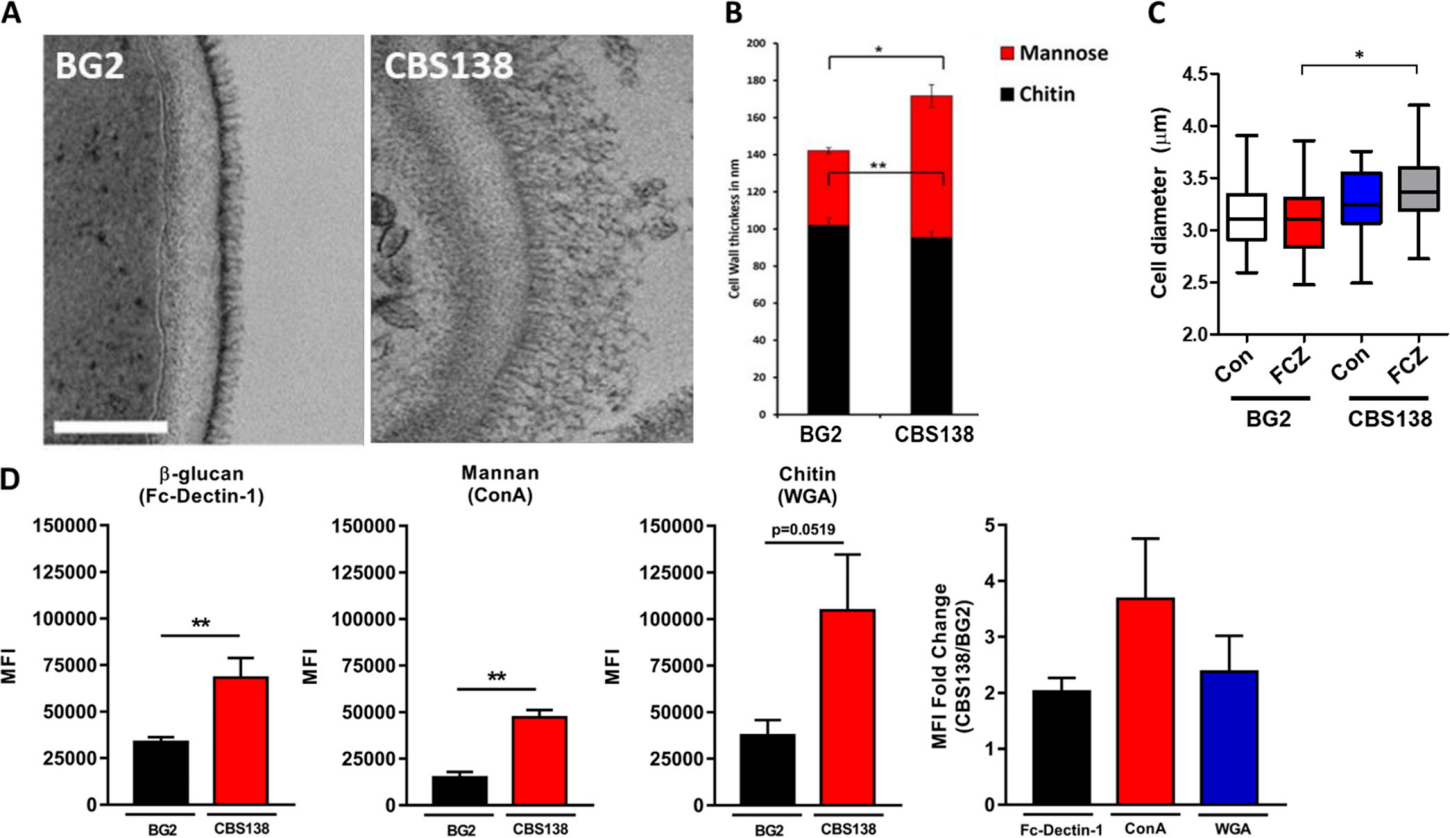
C. glabrata BG2 and CBS138 yeast cells are structurally distinct. (A) Transmission electron microscopy (TEM) comparison of BG2 and CBS138 cell walls. (B) TEM measurements of inner and outer cell wall thickness (indicated as chitin and mannose, respectively) for BG2 and CBS138 cell walls; *n* = 100 cells per strain. (C) Cell diameter was measured for BG2 and CBS138 yeast cells grown with and without fluconazole (MIC_50_). Data were generated in two independent biological experiments and represent 37 cell diameter measurements per experiment. (D) Mean fluorescence intensities (MFI) were determined by flow cytometry for BG2 and CBS138 cells stained for β-glucan (Fc-dectin 1), mannan (concanavalin A), and chitin (wheat germ agglutinin) exposure. MFI data represent the mean of 5 to 6 biological replicates plotted with SEM. The average fold change in MFI between strains was determined by dividing replicate CBS138 MFI values by BG2 MFI measurements for each respective cell wall component. Values based on high Fc-dectin 1 intensity/high β-glucan exposure gating from multimodal data only. *, *P* ≤ 0.05 between indicated groups. Statistical analyses were done by Mann-Whitney and Kruskal-Wallis tests.

The fungal cell wall is the first point of contact between yeast and host cells. Fungal glucan recognition by host cells can stimulate proinflammatory and fungal-clearing processes in immunocompetent hosts (34). However, previous work with C. glabrata demonstrated that it has some aptitude as an intracellular pathogen and does not provoke strong immune responses during systemic mouse infection, even persisting for weeks postchallenge in systemic organs (35, 36). Based on these observations, we investigated the cell wall structure and glucan exposure of CBS138 and BG2 using transmission electron microscopy (TEM), differential interference contrast (DIC) microscopy, and flow cytometry (Fig. 3).

The outer fibrillar layer of the cell wall in BG2 and CBS138 cells had marked differences in TEM images (Fig. 3A). The outer cell wall layer contained much longer fibrils in CBS138 cells than BG2, which appeared to have much shorter mannan fibrils densely packed together. The thickness of the outer cell wall was significantly larger for CBS138 at ~70 nm compared to just ~40 nm in BG2 (Fig. 3B). Both strains had a comparable inner cell wall thickness of ~90 to 100 nm.

Despite the differences in total cell wall thickness, overall cell diameter was, on average, between 3.1 and 3.3 μm for BG2 and CBS138 (Fig. 3C). In C. albicans, fluconazole treatment can induce mitotic collapse and the formation of larger, incompletely divided cells (37); therefore, we also investigated C. glabrata cell morphology and size after treatment with MIC_50_ fluconazole or dimethyl sulfoxide (DMSO) only (vehicle controls). Cell clustering was not evident after 4 h of fluconazole treatment, and cell size for BG2 remained similar to untreated cells (Fig. 3C). CBS138 did show evidence of increased cell diameter postfluconazole treatment, and cells were significantly larger than their fluconazole-treated BG2 counterparts (Fig. 3C).

The differences in cell wall structure highlighted by TEM prompted us to next investigate the carbohydrate exposure profile for each strain. We thus examined β-1,3-glucan, mannan, and chitin exposure using flow cytometry. BG2 had ~4-fold-lower fluorescence intensity for mannan staining with concanavalin A than CBS138 (Fig. 3D), which is consistent with the reduced thickness observed for the outer cell wall by TEM (Fig. 3A and B). Interestingly, BG2 also had reduced staining fluorescence intensity for chitin (wheat germ agglutinin [WGA]) and β-1,3-glucan (Fc-dectin 1) than CBS138, which were reduced by ~2.5- and 2-fold, respectively (Fig. 3D).

### Subtle differences in innate immune interactions and virulence between BG2 and CBS138.

**FIG 4:**
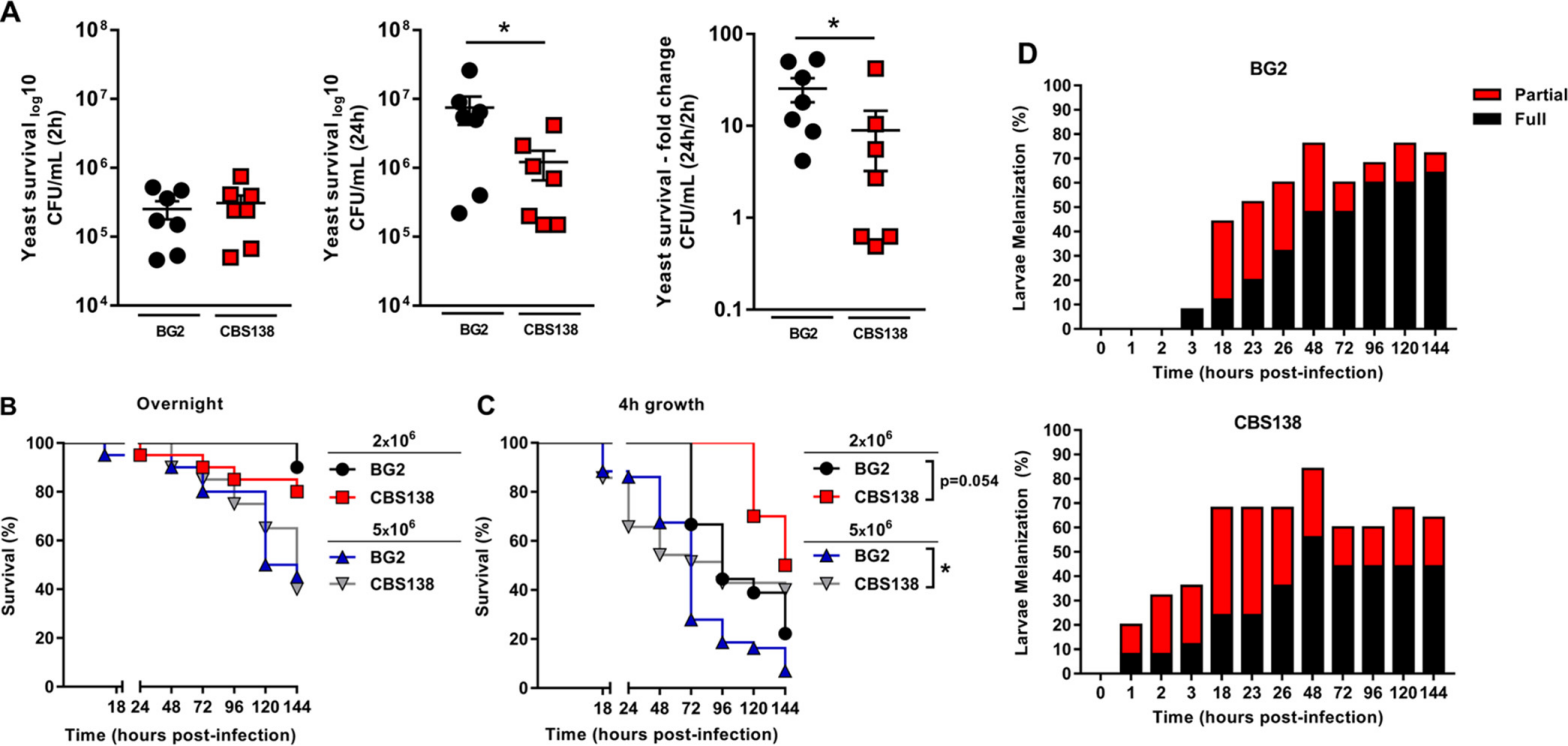
BG2 and CBS138 yeast cells have differences in survival during BMDM challenge and dose-dependent interactions with G. mellonella larvae. (A) BMDMs were challenged in technical duplicate at an MOI of 1:2 C. glabrata cells. Internalized yeast cells were determined at 2 and 24 h postchallenge. Data represent the CFU/mL of live yeast recovered by plating at 2 and 24 h following BMDM interaction and the fold change of yeast survival between these time points. The mean and SEM are indicated by the line and whiskers on each plot; *n* = 7 biological replicates per group for macrophage yeast survival. *, *P* ≤ 0.05 between indicated groups. Statistical analyses were done by Mann-Whitney. (B and C) G. mellonella larvae were injected with 2 × 10^6^ or 5 × 10^6^ BG2 and CBS138 yeast cells grown overnight (B) or for 4 h (C). Survival was monitored for up to 144 h postinfection. (D) Melanization of G. mellonella larvae infected with 5 × 10^6^ BG2 and CBS138 yeast cells was monitored during the survival study shown in panel C. Melanization was scored as partial or fully melanized as indicated in Fig. S4 in the supplemental material. *, *P* ≤ 0.05 between indicated groups. Statistical analyses were done by Kaplan-Meier. Data represent three independent experiments; *n* = 10 to 45 larvae per group for survival and melanization of G. mellonella.

We hypothesized that the differences in cell wall carbohydrate exposure observed in CBS138 and BG2 cells would affect macrophage recognition, fungal killing, and virulence. To test this hypothesis, we first challenged bone marrow-derived macrophages (BMDMs) with yeast at a multiplicity of infection (MOI) of 1:2 and assessed internalized yeast at 2 and 24 h postchallenge. Despite the significant differences in carbohydrate exposure between BG2 and CBS138 observed by cytometry, equivalent numbers of cells from both strains were taken up by BMDMs within 2 h (Fig. 4A). However, significantly more BG2 cells were recovered at 24 h postchallenge than CBS138 cells. In total, ~15-fold more cells were recovered for BG2 at 24 h post-BMDM challenge compared to ~7-fold more cells for CBS138 (Fig. 4A).

We next used the larval G. mellonella model to assess systemic virulence for stationary and actively dividing cells for each strain. Infection inocula were prepared from cells from each strain that were either grown overnight to stationary phase or back diluted and grown for a further 4 h. In general, G. mellonella survived at higher rates when infected with overnight-grown cells from BG2 and CBS138 compared to 4-h-grown cells, suggesting a mild attenuation in virulence from overnight culture conditions (Fig. 4B and C). While these data differ from a previous study that demonstrated that older cells were more virulent than younger generations (38), we believe these observed differences may be due to a mix of different generations in our overnight populations versus the sorted single generation used in the other work.

The virulence of overnight-grown cells was similar for BG2 and CBS138 based on G. mellonella survival (Fig. 4B). However, there were significant dose-dependent differences in virulence between the two strains when cells were diluted and grown for 4 h prior to infection (Fig. 4C). At a lower dose of 2 × 10^6^ cells per larvae, BG2 killed G. mellonella larvae from 72 h compared to 120 h for CBS138. G. mellonella survival was significantly different when infected with 5 × 10^6^ BG2 cells versus CBS138. Although initial larval death followed a similar timeline for both strains, G. mellonella survival with BG2 was capped at 5% compared to 40% survival with CBS138 challenge. G. mellonella melanizes, shifting from pale cream to gray and black, as infection progresses (Fig. S4). Surprisingly, G. mellonella larvae infected with CBS138 began to melanize as early as 1 h postinfection versus 3 h for BG2-infected larvae (Fig. 4D). However, by the end of the infection study, most BG2-infected larvae were fully melanized compared to approximately half (~45.5%) of CBS138-infected larvae (Fig. 4D), which correlates with larval death. Thus, both strains demonstrate important differences in virulence potential depending on dose and growth condition.

### Strain background influences virulence for nutrient sensing and cell wall-associated genes.

**FIG 5:**
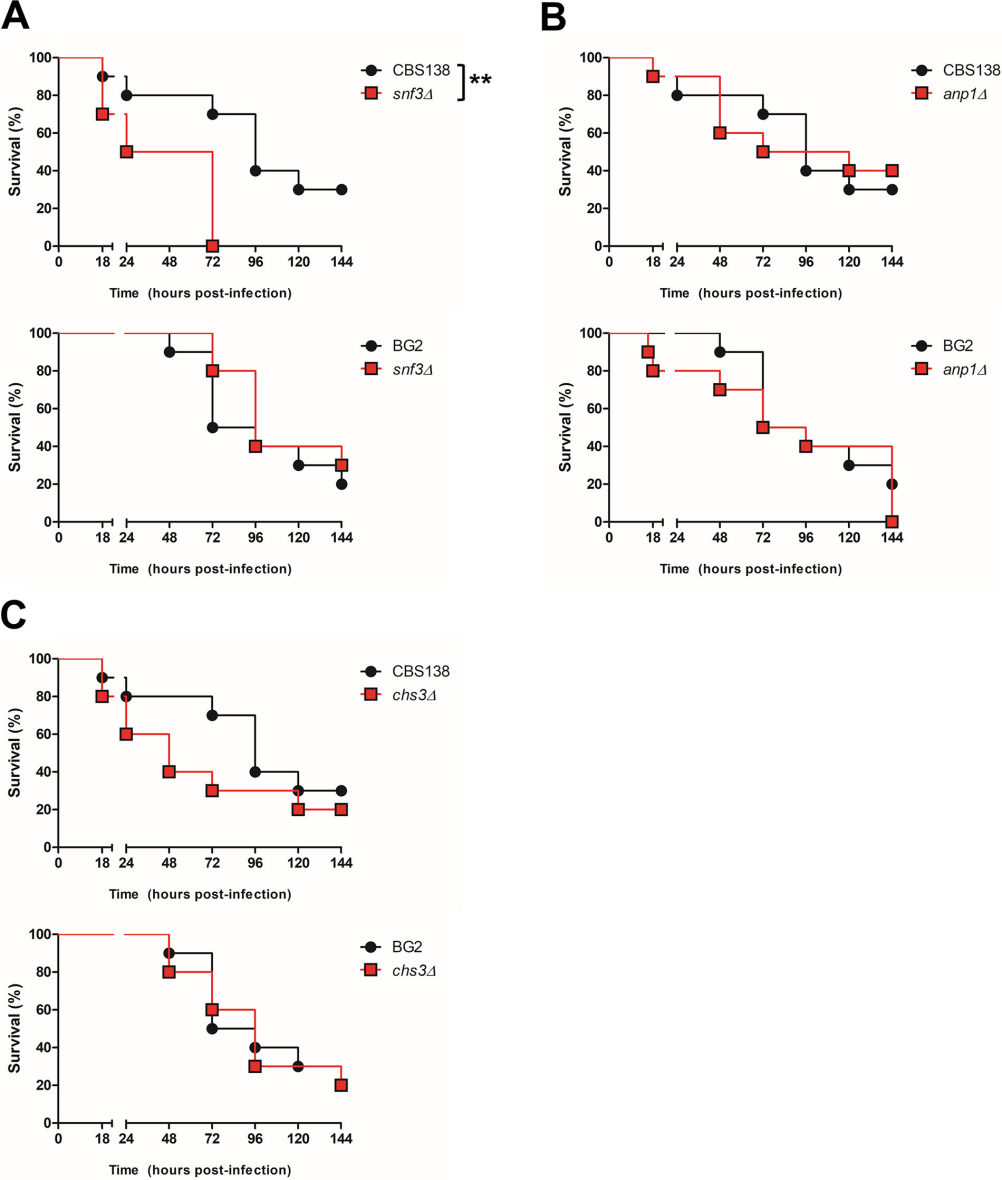
Strain background influences virulence for nutrient sensing and cell wall-associated genes. G. mellonella were infected with 5 × 10^6^ CBS138 or BG2 parent or *snf3*Δ (A), *anp1*Δ (B), or *chs3*Δ (C) cells. Survival was monitored for 144 h postinfection. Data shown are from one representative biological experiment with *n* = 10 larvae; three independent experiments were performed per mutant strain. **, *P* < 0.01; Kaplan-Meier statistical analysis was performed in IBM SPSS.

Our earlier findings highlighted differences between CBS138 and BG2 both in growth rate based on carbon source availability (Fig. 1) and in general cell wall architecture (Fig. 3). Metabolism and the cell wall play important roles in drug resistance and virulence in other fungal species, particularly C. albicans. Thus, we set out to determine how mutations in genes important for glucose sensing and cell wall architecture influenced virulence in each strain background using a G. mellonella infection model (Fig. 5).

One notable difference between these two C. glabrata reference strains was their carbon utilization and growth profiles for mannose and fructose (Fig. 1). *SNF3* encodes a transporter-like sensor for glucose, mannose, and fructose in S. cerevisiae; therefore, we investigated the role of *SNF3* in C. glabrata virulence in each of the strain backgrounds. Surprisingly, the CBS138 *snf3*Δ strain was significantly more virulent than its parent strain (*P* < 0.01) (Fig. 5A), while the BG2 *snf3*Δ mutant was not discernibly different in virulence from its respective parent (Fig. 5A). These results suggest that the metabolic differences between CBS138 and BG2 observed by Omnilog profiling play a biologically meaningful role during infection and further hint at important genetic rewiring between these strains.

In addition, we investigated the role of two cell wall-related genes, *ANP1* and *CHS3*, in each C. glabrata strain. *ANP1* is thought to play a role as a mannosyltransferase, and previous work has shown that deletion of this gene in CBS138 led to hypervirulence in a systemic mouse model of infection (39). Prior work with CBS138 *chs3Δ* has shown that this mutation leads to mannan or outer cell wall thickening via an unknown salvage pathway (40). In G. mellonella, infection with *anp1*Δ in either the CBS138 or BG2 background led to an early decrease in G. mellonella survival compared to parent strains, but overall, there was no significant difference in virulence (Fig. 5B). Similarly, CBS138 *chs3Δ* had a pronounced effect on early G. mellonella survival rates (30% survival versus 70% survival for CBS138 at 72 h) (Fig. 5C). However, BG2 *chs3Δ* had no defect in virulence compared to its parent strain (Fig. 5C). These data and the differences observed in cell wall architecture between these strain backgrounds (Fig. 3) suggest that the genetic network underpinning cell wall biogenesis has poorly understood strain-dependent plasticity that may affect drug resistance and clinical outcomes and thus requires further study.

## DISCUSSION

C. glabrata is a human fungal commensal and pathogen that can cause both superficial and life-threatening infections in immunocompromised people. It is closely related to S. cerevisiae yet causes significant morbidity and mortality in the clinic. C. glabrata pathogenesis is not well understood, with only a limited number of genes linked to virulence (27, 41). In part, this is due to the small number of existing *in vivo* studies and difficulty of replicating human disease in murine infection models. Most existing knowledge stems from studies with two key reference strains, CBS138 and BG2. With this work, we described important phenotypic differences that impact metabolism and virulence-related processes, which highlight the need to consider intraspecies phenotypic variation when translating strain-specific pathogenesis data to species-level conclusions.

Yeasts utilize carbon sources by a strict order of preference, which is enforced by the sensor/receptor repressor (SRR) pathway. Together with glucose, mannose and fructose belong to the top three most preferred sugars for yeast metabolism (33). In S. cerevisiae, these sugars have similar sensing and transport mechanisms via the SRR pathway. These three preferred sugars are sensed by Snf3 and Rgt2 and can be transported into the cell by all major yeast glucose transporters. The ability to metabolize multiple different carbon sources can influence important virulence factors such as the ability to form biofilms (41, 42). C. glabrata can sense a broad range of nutrients within host niches, and we observed that this nutrient sensing and utilization is divergent in the reference strains BG2 and CBS138. Further, deletion of *SNF3* enhanced virulence in the CBS138 background, suggesting that sugar nutrient sensing via *SNF3* negatively modulates virulence in this strain. Nutrient sensing and utilization mechanisms likely play a role in the switch from commensalism to emergence of infection, as in the human host, the fungal cell can proliferate under both nutrient-rich and nutrient-poor conditions. It is this adaptation to different environments that has been one of the keys to success of C. glabrata as a pathogen.

In our study, we found important metabolic differences between two C. glabrata reference strains that could impact experimental outcomes. Specifically, we determined that while BG2 could use methionine and tryptophan as nitrogen sources, CBS138 had significant defects utilizing these amino acids (Fig. 1 and 2). Amino acid metabolism plays an important role in virulence for multiple fungal pathogens and is particularly well studied in C. albicans and C. neoformans. Methionine interacts with G protein-coupled receptors, CaGpr1 and CnGpr4, inducing receptor internalization and signaling in both C. albicans and C. neoformans (43, 44). In C. albicans, methionine metabolism has been linked to PKA signaling and hyphal formation (45), which is a key virulence factor. C. neoformans methionine permeases CnMup1 and CnMup3 support capsule formation (46), and tryptophan induces Cn*MUP1* and Cn*MUP3* expression. Loss of Ca*CSH3*, which is involved in nitrogen source utilization, impairs tryptophan use and virulence (47). Further, amino acid metabolism is known to be important for C. albicans survival after phagocytosis by immune cells (48–50). It remains to be determined whether amino acid-induced GPR and PKA signaling is conserved in CBS138. However, defects in tryptophan and methionine metabolism, such as those observed in CBS138, could be detrimental to survival *in vivo* compared to BG2. For instance, BG2 and CBS138 had similar intracellular cell counts 2 h postchallenge with BMDMs, yet significantly fewer CBS138 cells were recovered at 24 h postchallenge than BG2 (Fig. 4A). Future studies are necessary to directly link changes in intracellular survival to metabolic differences between these strains.

Metabolism also plays an important role in virulence by producing precursors important for stress resistance and antifungal resistance and by modulating cell wall architecture and plasticity (48–51). The cell wall is a critical facilitator for host-pathogen interactions and is dynamically modified based on environmental and nutritional cues (51–53). Modifications in mannosylated proteins or their abundance as well as to immunomodulatory carbohydrates, including β-1,3-glucan and chitin, can promote immune evasion, tolerance, or severe inflammation (52, 54). In our work, we observed significant differences in cell wall architecture between BG2 and CBS138 by TEM that correlated with carbohydrate staining by flow cytometry, where CBS138 exposed more mannan, β-1,3-glucan, and chitin in its cell wall than BG2 (Fig. 3A, B, and D). Despite elevated β-1,3-glucan exposure, CBS138 had similar levels of intracellular yeast cells present in BMDMs at 2 h compared to BG2 (Fig. 4A). This result suggests that both strains had no significant differences in early recognition by BMDMs, leading to similar levels of phagocytosis. It is also possible that altered cell wall composition is not a significant factor in C. glabrata-macrophage interactions since caspofungin treatment studies have shown that drug-induced changes in the C. glabrata cell wall did not impact yeast phagocytosis by and survival in J774 macrophages (55). Further study is required to establish the role of the C. glabrata cell wall in determining innate immune interaction outcomes.

However, while BG2 was more virulent during G. mellonella infection, larvae infected with CBS138 melanized at earlier time points than larvae infected with BG2 (Fig. 4C and D). These melanization data suggest that larvae mounted a rapid response to CBS138 infection, which may account for the later differences in virulence between the two strains. These differences in virulence and melanization timelines are also consistent with our cell wall cytometry data, specifically the data that indicated that BG2 exposed smaller amounts of all three major cell wall carbohydrates than CBS138 (Fig. 3B and C). Interestingly, G. mellonella larvae infected with cell wall mutants *anp1*Δ (BG2 and CBS138 backgrounds) and *chs3*Δ (CBS138 background only) also induced slightly earlier larval death than parent strains, further suggesting that the cell wall modulates early host responses that influence disease outcomes. G. mellonella has several β-1,3-glucan binding proteins and C-type lectin receptors that prime hemocyte responses and induce antimicrobial peptide production (56, 57). Thus, these receptors may be able to detect the differences in exposed carbohydrates between strain cell walls, leading to altered early immune responses. It was previously reported that heat-inactivated C. glabrata cells are avirulent; thus, cell wall and carbohydrate recognition differences are likely part of a polygenic, active C. glabrata process required for disease (58). Interestingly, the expression of a key cell wall-associated virulence factor, the adhesin *EPA1*, is heterogenous in the BG2 background but not in CBS138 and may play a role in differences in virulence (22). Therefore, more work needs to be done to address the role of subpopulation phenotypic heterogeneity in driving C. glabrata pathogenesis.

Our investigation has identified important similarities in metabolism and host interactions between BG2 and CBS138 but has also noted key differences whose implications for disease are not well understood. In particular, our infection study with *snf3*Δ and *chs3*Δ produced significant yet different results in CBS138 and BG2 that highlight how intraspecies heterogeneity in genetic and phenotypic determinants are biologically meaningful in the context of virulence. Therefore, it is vital to consider these potential limitations when conducting virulence studies in one strain background in order to draw robust conclusions about essential C. glabrata disease processes.

## MATERIALS AND METHODS

### Fungal strains and growth conditions.

Candida glabrata strains BG2 and CBS138/ATCC 2001 (23, 28, 29) were maintained by weekly subcultivation in 2% glucose, 2% bacto-peptone, 1% yeast extract, and 2% bacto-agar (YPD) plates at 37°C. Before each experiment, yeast cells were conditioned overnight in MOPS (morpholinepropanesulfonic acid)-buffered liquid RPMI 1640 medium (2% glucose, pH 7) (Sigma; catalog no. R6504). Yeast cells were grown overnight in 5 mL RPMI 1640 followed by a further 4 h growth in 10 mL or 100 mL RPMI 1640 with antifungals (MIC_50_ concentration) or DMSO only as specified on each experimental method.

### Macrophage yeast survival.

Bone marrow-derived macrophages (BMDMs) were isolated from the femurs and tibias of male 12-week-old C57BL/6 mice. Mice were randomly selected from in-house breeding colonies housed under specific-pathogen-free conditions. Mice were not subjected to any regulated procedures prior to cervical dislocation and femur removal in accordance with ethical regulations approved by the University of Aberdeen Animal Welfare and Ethical Review Body. BMDMs were maintained and differentiated in Dulbecco’s modified Eagle’s medium (DMEM; Sigma) supplemented with 10% heat-inactivated fetal calf serum (Gibco), 15% L929 cell-conditioned medium, 1% l-glutamine, and 1% penicillin-streptomycin (Sigma) (59). For macrophage interaction studies, 3 × 10^4^ BMDMs were plated on flat-bottom 96-well plates and incubated overnight at 37°C and 5% CO_2_. BG2 and CBS138 wild-type cells were grown overnight in RPMI 1640 and counted, and 1 × 10^7^ cells were grown for a further 4 h with <1% DMSO in 10 mL RPMI 1640 (2% glucose, pH 7) at 37°C. Yeast cells were then washed with phosphate-buffered saline (PBS) and added to the 96-well plates in duplicate wells at a multiplicity of infection (MOI) of 2:1 (yeast cells to macrophages). Two hours postchallenge, the supernatant was removed, each well was washed with DMEM, and new medium was added to remove unengulfed yeast cells. For the 2-h time point, 100 μL of 0.02% chilled SDS was added to each well, and its contents were scraped, serially diluted, spotted on YPD agar plates, and incubated at 37°C. After 24 h, the CFU/mL was determined. The same BMDM lysis and yeast recovery procedure was performed at 24 h.

### Galleria mellonella infection, survival, and melanization.

G. mellonella larvae were purchased from Livefood UK and stored in wood shavings in the dark at room temperature prior to experimentation. BG2 and CBS138 wild-type cells were grown overnight in RPMI 1640 medium (2% glucose, pH 7) at 37°C, counted, and diluted to 1 × 10^8^ yeast cells in 100 mL RPMI 1640 and grown for a further 4 h. Cells were washed and resuspended in sterile PBS. G. mellonella larvae (~250 mg weight) were randomly allocated into groups of 25 and infected in the last left proleg with 2 × 10^6^ or 5 × 10^6^ C. glabrata cells—from overnight and further 4-h-grown cultures—in a 50 μL suspension using a U-100 30G BD microfine syringe. A control group of 25 larvae received an injection of 50 μL sterile saline only. Larvae were incubated at 37°C in the dark, and survival and melanization were assessed daily for a period of 6 days. Larvae were considered partially melanized when their natural color had been visibly altered; however, they still did not present a fully darkened body (see Fig. S1 in the supplemental material). Larvae were considered fully melanized when their color had been completely altered to a dark gray/brown pigmentation (Fig. S1).

To assess mutant virulence, parent and mutant strains were grown overnight at 37°C in RPMI 1640 medium, washed twice, and resuspended in PBS. Groups of 10 G. mellonella larvae were inoculated with 5 × 10^6^ C. glabrata cells in a 50-μL suspension as described above, and survival was monitored over 6 days. Three independent biological experiments were performed for each mutant and parent strain.

### Flow cytometry and microscopy analysis.

Cells were inactivated overnight in 50 mM thimerosal (Sigma-Aldrich), washed three times with PBS, and counted by hemocytometer. To analyze cell wall carbohydrate exposure, 2.5 × 10^6^ cells were stained with Fc-dectin 1 (kindly gifted by Gordon Brown, MRC-CMM) and goat anti-human IgG antibody conjugated to Alexa Fluor 488 (Invitrogen), wheat germ agglutinin (WGA) conjugated to Alexa Fluor 680 (Invitrogen), and concanavalin A (ConA) conjugated to Texas Red (Invitrogen). Data were acquired for a minimum of 20,000 events on the Attune NxT (Thermo Fisher) and analyzed using FlowJo v10 software (TreeStar Inc., Ashland, OR). Multimodal data were analyzed based on Fc-dectin 1 gating, with mean fluorescence intensities calculated for all three fluorophores based on “high” Fc-dectin 1 gates (Fig. S3). For cell size diameter determination, BG2 and CBS138 were grown for 4 h at 37°C and 200 rpm in RPMI containing 1% DMSO (controls) or MIC_50_ concentrations of fluconazole for each strain. Cells were then inactivated as described for flow cytometry analyses and imaged on a Zeiss Axioplan 2 microscope with phase contrast. Images were captured on a Hamamatsu C4742-95 digital camera (Hamamatsu Photonics) and recorded and analyzed for cell diameter using Zeiss Zen software (Oberkochen, Germany).

### Omnilog.

Cells were grown overnight in soybean casein digest (SCD) medium at 37°C followed by two washes in sterile distilled water and diluted to an optical density at 600 nm (OD_600_) of 0.5 (cells added to PM medium at a final OD_600_ of 0.01), followed by incubation in Omnilog at 37°C for 48 h. Analysis was performed using the R package opm (59), the raw kinetic data were imported from the Omnilog station, and the opm workflow was followed (Tables 1 and 2).

**TABLE 1:** Plate setup for Omnilog

PM stock solution	Amt of stock solution (mL) in:	
	PM01 and PM02	PM03
IFY-0	20	40
SC medium		
Dye mix D	0.32	0.64
d-Glucose		1.5
PM additive	2	4
Cells (at OD_600_ of 0.5)	0.5	1
Sterile water	1.18	0.86
Total	24	48

**TABLE 2:** Additives for PM Omnilog plates

PM additive	Amt (mM)
PM1 and PM2	
l-Glutamic acid monosodium	60
Potassium phosphate monobasic anhydrous (pH 6.0)	60
Sodium sulfate	24
PM3	
Potassium phosphate monobasic anhydrous (pH 6.0)	60
Sodium sulfate	24

### Statistical analyses.

Statistical analyses were performed using GraphPad Prism v7.0 software (GraphPad Software, La Jolla, CA, USA), and IBM SPSS Statistics v27.0 (IBM Corp., Armonk, NY, USA). The macrophage yeast survival and flow cytometry analyses were done by Mann-Whitney and Kruskal-Wallis tests, and the G. mellonella survival was analyzed by Kaplan-Meier. A *P* value of <0.05 was considered to be significant, and the results are shown as mean ± standard error of the mean (SEM).

### Generation of C. glabrata deletion strains.

Strains used and generated in this study are listed in Table S2. The *TRP* cassette was amplified from genomic DNA with complementary up- and downstream sequences for each ORF, (Table S1 for primers used), using the following conditions for a 50-μL reaction mixture: 1× *Taq* buffer, 0.2 μM dNTPs, 0.5 μM each primer, 1 μL Taq-polymerase, and plasmid DNA at 93°C for 3 min, with 30 cycles of 93°C for 30 s, 50°C for 30 s, and 72°C for 2 min with 10 min at 72°C. The fragments were gel purified in 0.7% agarose gels, and the final deletion construct was purified via ethanol precipitation. The final deletion cassette was transformed into C. glabrata parental strains BG2 and CBS138 via electroporation (60). The correct transformants were selected for growth on synthetic complete medium lacking tryptophan (SC-trp). Three independent transformants of each strain were collected and sequence verified.

### Transmission electron microscopy.

Overnight liquid cultures of BG2 and CBS138 were grown in 10 mL YPD. Prior to high-pressure freezing, cells were concentrated into a paste and placed between the sides of a small copper holder. The sandwich was then frozen in a high-pressure freezer (high-pressure freezer; HPM Live μ). The freezing achieved near instantaneous cessation of cellular processes and locked the cell structure into a vitreous ice matrix. Following freezing and in liquid nitrogen temperatures, the cells were placed into chemical fixatives; mixtures of glutaraldehyde (1 to 3% glutaraldehyde) or osmium tetroxide (2 to 4%), both diluted in 100% acetone and containing 0.05 to 0.1% uranyl acetate, were used during the substitution phase of preparation. Following incubation at −80°C for several days, the sample was allowed to warm very slowly. As the sample warms, the fixative-solvent mixture replaced or substituted for the cellular water, fixing and dehydrating the cells simultaneously. Once warmed to room temperature, dehydration was completed in several changes of 100% acetone and embedded in resin. Thin sections were cut and stained. Images were acquired on a Jeol JEM-1400 transmission electron microscope.
