# Supplementary material for: The Candida glabrata Parent Strain Trap: How Phenotypic Diversity Affects Metabolic Fitness and Host Interactions
Source: Microbiol Spectr. 2023 Jan 12;11(1):e03724-22. doi: 10.1128/spectrum.03724-22 (PMC9927409; doi:10.1128/spectrum.03724-22)
Supplement: Supplemental file 3 — Fig. S1 to S4. Download spectrum.03724-22-s0003.pdf, PDF file, 1.1 MB [file spectrum.03724-22-s0003.pdf]

Carbon usage

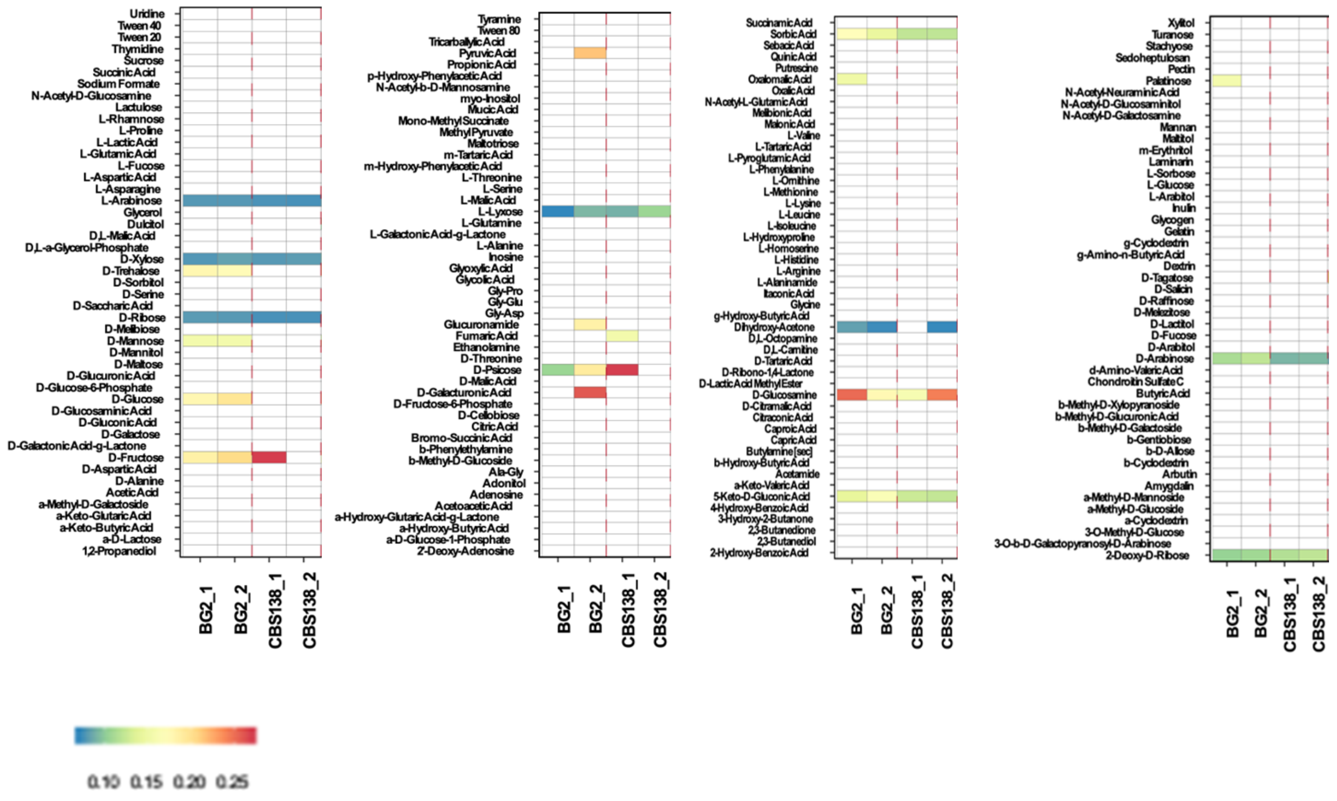

Figure S1. Complete Omnilog carbon utilization dataset for BG2 and CBS138.

Nitrogen usage

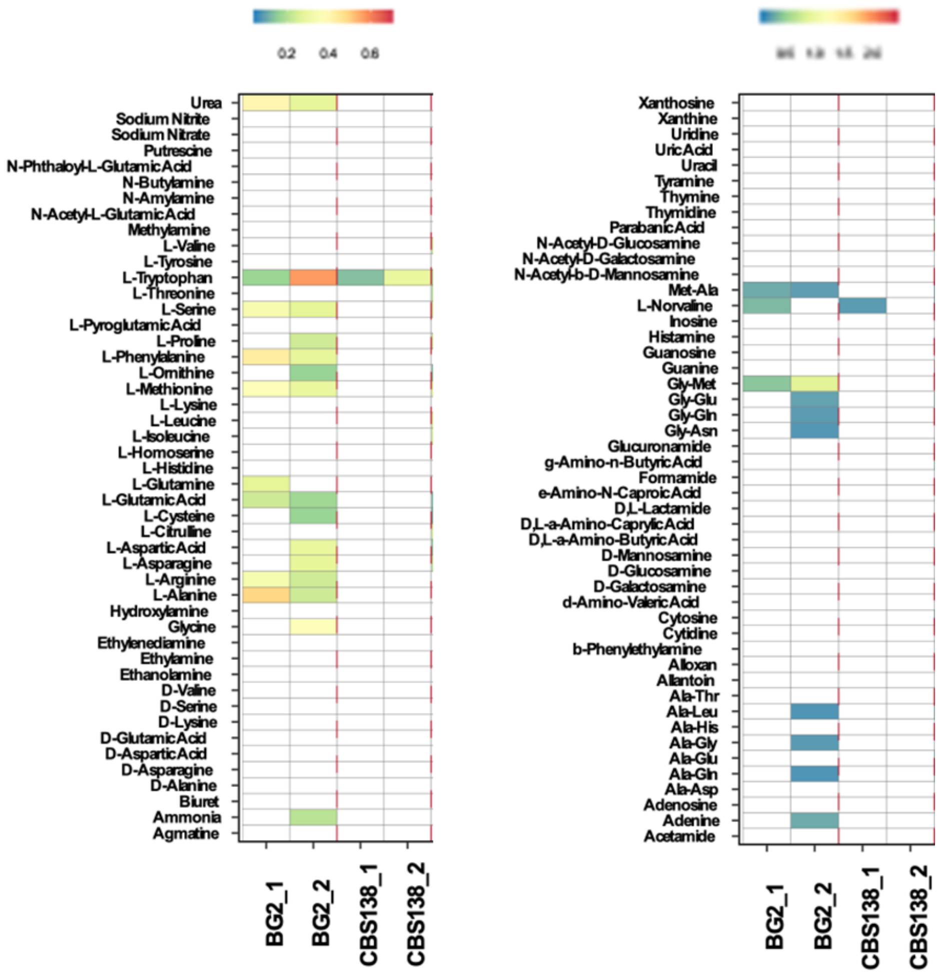

Figure S2. Complete Omnilog nitrogen utilization dataset for BG2 and CBS138.

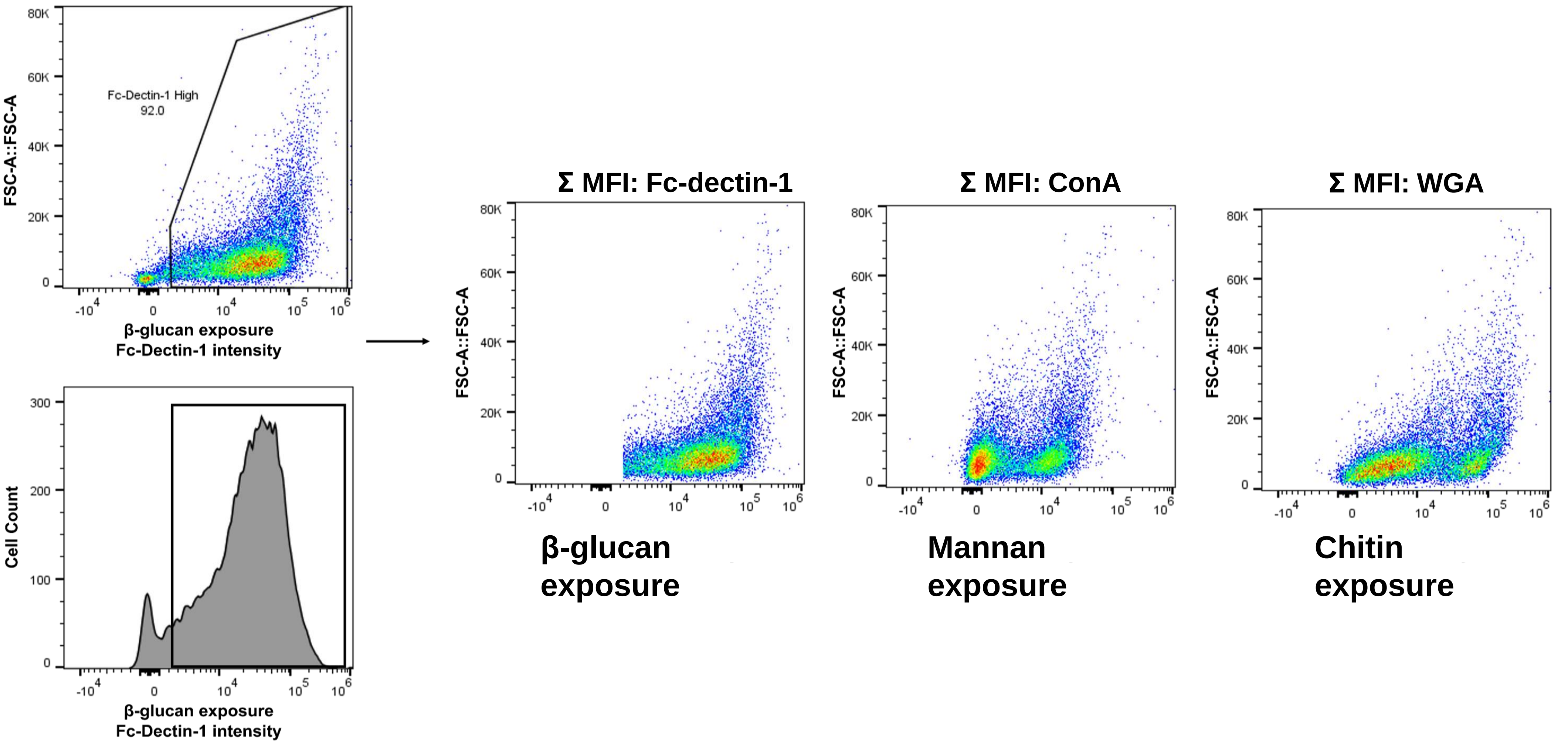

Figure S3. Flow cytometry gating strategy for multimodal datasets. C. glabrata multimodal populations were gated on the basis of the scatter plot (FSC-A) or histogram (cell count) and β-glucan expression (Fc-Dectin-1). Mean Fluorescence Intensity of each cell wall marker was established in reference to Fc-Dectin-1 high exposure levels.

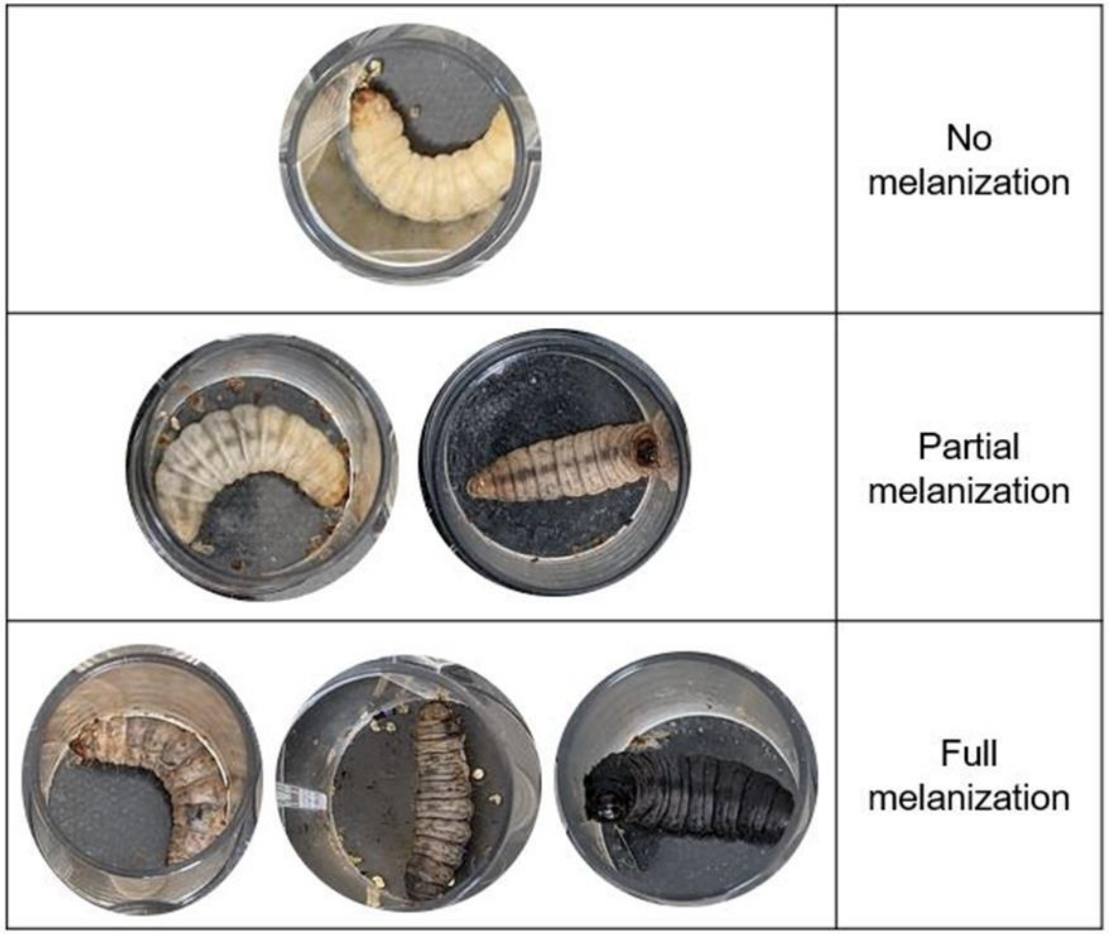

Figure S4. *G. mellonella* melanization scoring guide. *G. mellonella* larvae were considered non-melanized when normal cream color was maintained. Larvae were considered partially melanized when the body color changed slightly either along the whole body or in sections of it and/or a darker line was visible. Larvae were considered fully melanized when the body color had fully changed to darker tones. Dead larvae were also included in the assessment as fully melanized (black).
